# Evaluating the Intention and Behaviour of Private Sector Participation in Healthcare Service Delivery via Public-Private Partnership: Evidence from China

**DOI:** 10.1155/2020/5834532

**Published:** 2020-01-16

**Authors:** Jie Yang, Lingchuan Song, Xiaoyi Yao, Qian Cheng, Zichao Cheng, Ke Xu

**Affiliations:** ^1^School of Management Engineering, Shandong Jianzhu University, Jinan 250101, Shandong, China; ^2^School of Business, University of Leicester, Leicester LE3 5EB, UK; ^3^Department of Cardiology, Beijing An Zhen Hospital, Capital Medical University, Beijing Institute of Heart Lung and Blood Vessel Disease, Beijing 100069, China

## Abstract

Private sector participation in the healthcare market via public-private partnership (PPP) could be considered an available approach to narrow down the medical resource gap and improve the operational efficiency of healthcare facilities. Accordingly, this study aims to examine the influence and relative importance among critical factors for the intention and behaviour of the private sector towards participation in Chinese healthcare market (CHM) via PPP. We defined five hypotheses from previous literature and built a theoretical model based on modified theory of planned behaviour. Then, covariance-based structural equation modelling was applied to analyse the questionnaires provided by 248 respondents from construction companies, real estate developers, pharmaceutical companies, private hospitals, asset management companies, and medical industry property investment companies in China. Results indicated that attitude towards behaviour (*β* = 0.466, *P* < 0.001), subjective norm (*β* = 0.167, *P* < 0.05), perceived behavioural control (*β* = 0.231, *P* < 0.01), and facilitating conditions (*β* = 0.305, *P* < 0.001) are positively significant to behavioural intention; behavioural intention also shows a strong linkage with behaviour (*β* = 0.931, *P* < 0.001). Findings provide reference for governments and public authorities to exert additional efforts in implementing appropriate measures that will stimulate the private sector's motivation to participate in CHM via PPP.

## 1. Introduction

Chinese healthcare market (CHM) has rapidly developed with economic growth in recent decades [1]. CHM has been one of the most involved fields to draw investments and efforts from the public sector [2]. In 2009, China started its healthcare reform to establish basic medical and health system covering all residents from urban to rural areas [3]. Achievements were made during the process of reform; to illustrate, 93% of individuals were covered by insurance [4], but the imbalanced distribution of resources between public general and private hospitals remains severe. Statistics from National Health Commission (NHC) indicates that approximately 70% of medical service demand in China currently comes from primary communities, and tertiary public services account for 75.7% of total hospital beds and 85.8% of outpatient visits at the end of 2017 [5]. Traditional stereotyping predisposes people to seek services from tertiary hospitals rather than primary care or private facilities even for chronic noncommunicable diseases. The participation of private capital in the market of healthcare via public-private partnership (PPP) could be a feasible and sustainable approach to narrow down the gap between demand and supply of medical services effectively.

In general, public institutions possess the dominant position in the CHM [6], showing absolute advantages in equipment, technical knowledge, and highly educated personnel. Traditionally, benefits of public hospitals originate from financial subsidies and drug sales, resulting in overprescription and unnecessary procedures [7]. Moreover, the number of public healthcare services reflects a mismatch with the huge market demand. The excessive concentration of patients to public hospitals resulted in the emergence of the “rent dissipation” phenomenon, such as queues and scalped trading [8]. Thus, waste of resources leads to efficiency defect of the entire society and damages the sustainability of medical services in CHM.

Two typical routes exist for the implementation of private sector investment in the healthcare field, namely, privatisation and PPP. These two concepts must be distinguished. Privatisation refers to the transfer of ownership and responsibilities from public services to private capital; it brings competition and market mechanism [9]. Opponents believe that a paradox exists between privatisation and welfare economic theory. Healthcare as public good related to social responsibility should be equally accessible to all individuals without certain limitations [10]. Compared with privatisation, PPP was interpreted as a long-term contractual agreement between a public agency and a private sectoral entity, through which each sector shares the skills, assets, risks, and rewards in the delivery of a service and facility for use of the general public [11]. At the end of 2018, the debts of local governments in China reached 18.4 trillion yuan [12]; public authorities were under huge pressure to spend on social healthcare expenditures. Private capital involved in medical delivery via PPP could expand the source of funds, improve the operational efficiency of healthcare facilities [13], strengthen their own market competitiveness, and acquire opportunities for future investment. Emerging countries are considered a proper market in extensively promoting PPP to improve the quality of medical infrastructures [14]. Four representative types are applied for private sector investment in CHM via PPP modality, namely, build-operate-transfer (BOT), renovate-operate-transfer (ROT), transfer-operate-transfer (TOT), operation and management contracts (O&M) [15] (Table 1).

Despite PPP's substantial contribution to creating high capacity and quality of health system in China [15], challenges remain. Firstly, numerous private capital entities, including construction companies and property developers, lack technical knowledge and experience to operate healthcare facilities [16]. Secondly, considering certain differences among medical entities, setting appropriate key indicators to evaluate performance for private participators as payment basis is difficult [14]. Thirdly, private specialist clinics are still not allowed to establish property relation with public general hospitals in several core medical services owing to policy restrictions [17]. Finally, the value conflict between public welfare and profit seeking in healthcare product remains harsh [18].

These benefits and challenges will be difficult to ignore for CHM participators during the decision-making process. Thus, determining key factors for the intention of the private sector to participate in CHM via PPP modality is necessary. This study aims to examine the influential factors that affect the intention and behaviour of private sector involvement in CHM via PPP. Firstly, five hypotheses are defined by literature research based on modified theory of planned behaviour (TPB). Then, the questionnaire survey method is employed to investigate the intention and behaviour from private capital participation in CHM via PPP. Furthermore, we applied covariance-based structural equation modelling (CB-SEM) to analyse the data from questionnaire survey and subsequently drew the conclusion. Finally, the findings and limitations of this study were discussed.

This study could expand the research scope of intention and behaviour in CHM for the private sector and also develop an analytical framework as reference for other CHM stakeholders to make rational selections.

## 2. Literature Review

### 2.1. Critical Challenges in CHM

Three notable challenges in CHM are identified owing to the imbalance between demand and supply of high-quality diagnosis [19]. Firstly, doctors and patients were unsatisfied with the conditions of current practice [20]. Referral system remains absent in China [21], doctors in large-scale hospitals suffered from work overload and personal assaults [22, 23]. Approximately 95.66% of doctors were reported to consider that a mismatch exists between efforts and salaries [24]. In addition, healthcare practitioners held a tense relationship with patients. Several patients and their families even came into conflict with doctors to protest against the insufficient medical resources and expensive fees of drugs and treatments [25, 26]. Secondly, the phenomena of overprescription and overdiagnosis were common in CHM [21], especially in primary or secondary cares of rural areas [27]. On the one hand, public hospitals were allowed to profit from services and drugs to compensate for the low subsidies from governments [28, 29]. On the other hand, unnecessary diagnostic tests, drugs, and therapeutic interventions were regarded as defensive actions for doctors to avoid potential disputes [30]. Thirdly, the challenge is the resource shortage and waste of primary healthcare institutions in CHM [31–33]. Li et al. indicated that low reimbursement caps and limited coverage provided incentives for patients to overuse public hospitals and prevented primary medical institutions from providing first-contact care [34]. Researchers showed that a large room could be provided in the primary and secondary healthcare markets in rural China, which could be made available for private capital investment [32].

Furthermore, traditional economic theory believed that efficiency and fairness are two major aims that social managers must consider in initiating policies and intervening in the market [35]. In China, establishing an adequate healthcare system to ensure that each individual gains access to medical services was a remarkable feature for achieving fair opportunities and a manifestation of healthcare democracy [36]. However, Yuan et al. argued that less than 5% of the financial burden on medical expenses had been cut [37]. Most of the households in Mainland China still weakly resist expensive catastrophic or chronic diseases [38]; consequently, they must increase their savings as a precaution for the future burden of medical bills [39]. In addition, findings from Kuan and Chen suggested that the prohibitive expenditure on healthcare has severely limited family consumption in other daily activities [40].

An overwhelming trend requiring emphasis is ageing. Scholars considered that China has reached the Lewis turning point and has become an ageing society [41]. Statistics showed that 20% to 33% of individuals will be aged 65 years or older by 2050 [42]. Thus, this change in demographics will result in considerable levels of somatic disease and comorbidity [43]. The booming demand for healthcare services and pension cares from the elderly heightened the shortage of medical resources [44]. Moreover, Niu et al. argued that community-based primary healthcare generates poor performance, which scarcely met the satisfaction of the elderly [45]. Existing literature suggested an urgent need to increase the quality and quantity of CHM.

### 2.2. PPP in Healthcare Service Delivery

As a typical category of social infrastructures with increasing demand, healthcare was regarded as a suitable field for applying PPP [46]. European countries had a relatively universal application of this model to develop and deliver healthcare facilities [47]. Data from European PPP Expertise Center (EPEC) showed that healthcare was the largest PPP sector, with huge market transaction volume [48]. Scholars collected 49 PPP hospital cases from the UK and developed an analytical model to emphasise that PPP hospital could achieve value for money under stable political and economic circumstances [49]. However, considering the diversity of medical facilities and complexity of performance measurements, PPP in healthcare was also confronted with challenges [50]. A previous study from Italy suggested that irrational and ill-advised development could spoil the sustainability of PPP healthcare [14]. Torchia and Calabrò underlined a critical issue: compared with other fields, the health landscape had been taking a period of enormous change, including clinical technologies, models of care, and epidemiological trends; thus, the uncertainty was largely magnified [51].

In effect, four approaches for private sector participation in healthcare delivery via PPP were concluded by Barlow and his colleagues [47, 52]. Firstly, private entities provide medical facilities and nonclinical work without intensive cooperation. Secondly, a new project company is established for the purpose of operating clinical services [53]. The third model is concession, which would be tightly supervised by health authorities [54]. Finally, the private sector could deliver hospital services and primary care for individuals in their location [55]. These approaches have been used in numerous developed and emerging countries, especially in Europe.

Chinese and foreign scholars hold a consensus that PPP is a combination of strength from public and private sectors for the fulfilment of cost efficiency and social policy [56, 57]. Since 2014, the new round of PPP tide has rapidly emerged and has profoundly contributed to expanding the supply of urban infrastructures in China [58, 59]. Akin et al. considered that compared with municipal engineering or transportation, CHM embraces relatively low marketisation [60]. PPP could be seen as a part of China's healthcare reform to release the financial burden of medical facilities and introduce advanced management concept in CHM [61, 62]. In Guangdong, an empirical study was launched using grey relational analysis to build a research model and compare the resource configuration and service ability among “Chaonan Minsheng Hospital” (which is a certain PPP hospital in Guangdong), normal public hospitals, and private hospitals. Accordingly, the PPP hospital has been found to have better resource allocation and service quality than the other two types [63]. In addition, Zhang concluded several applicable forms in which the private sector could participate in CHM via PPP and then emphasised that attention should also be paid to cultivating the medical soft power, including the construction of key disciplines and training of general practitioners [64].

Based on previous literature, no relevant research on the intention and behaviour of private sector participation in CHM exists, especially via PPP. Thus, the question below must be answered:

What are the influential factors and to what extent do they affect the intention and behaviour of private sector involvement in Chinese healthcare service delivery via PPP?

## 3. Theoretical Model and Hypotheses

### 3.1. Theoretical Model Based on Modified TPB

TPB was developed by Ajzen in 1988 [65] as an expansion of theory of reasoned action (TRA) [66]. Ajzen claimed that personal behaviour is affected by voluntary and various factors. TRA could not reasonably explain involuntary behaviour. Thus, Ajzen put forward three indicators to analyse the decision-making process and individual action, namely, attitude towards behaviour (AB), subjective norm (SN), and perceived behavioural control (PBC). These three factors could affect the behavioural intention (BI) and then behaviour (B) [65].

In 1995, Taylor and Todd built an extended model, which introduced perceived usefulness as a critical factor based on the TPB model [67]. These scholars believed that the extended model could compensate the measurement of a certain group's feeling towards a concept.

TPB was originally proposed to investigate a user's willingness to accept a certain technology. With its wide prevalence, TPB was also applied in numerous fields that analyse human behaviour [68, 69]. Several studies were implemented on the BI of the private sector. East was the earliest researcher who used behaviour theory in the field of private investment [70]. Paetzold and Busch developed a framework based on TPB to understand the decision-making process of the private sector towards sustainable investment [71]. Malaysian scholars applied a modified TPB model, which explained the effect of the private sector's intention and attitude to participate in socially responsible investments [72]. In addition, TPB could be considered a framework to evaluate the willingness of private capital to invest in normal PPP projects and analyse the connection between the private sector and PPP performance [73, 74]. In CHM, researchers applied TPB to examine the patients' willingness to seek treatment [75], consuming intention on medical products and nurturing personal health behaviour [76, 77].

**Figure 1:**
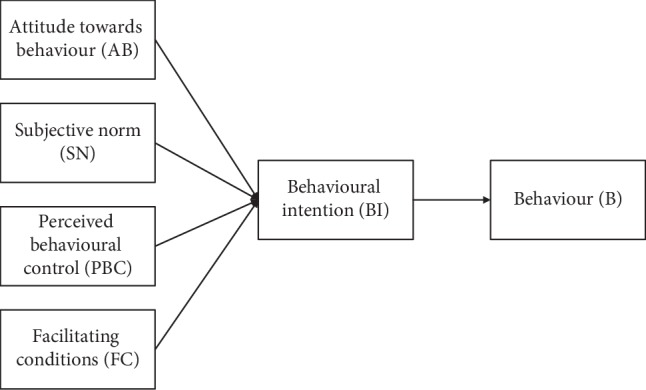
Structural model based on the modified TPB.

Considering the different approaches of PPP modality and policy circumstance of CHM, the objective environment and competitive status should be emphasised whilst the private sector participates in CHM via PPP. Thus, to build a research model with good explanatory effect, facilitating conditions (FC) as a main indicator are identified and introduced in our modified TPB model from Venkatesh's finding [78]. In this research, AB, SN, PBC, FC, BI, and B are regarded as latent variables in the structural model (Figure 1).

### 3.2. Hypothesis Development

#### 3.2.1. Determinants of Private Sector's BI to Participate in CHM via PPP

AB is defined as feeling on the target behaviour and could be regarded as individuals' affective reaction on the outcome of assuming a particular behaviour [71, 78]. Boyne considered that the private sector is driven by profit motives and self-interest [79]. Thus, investment benefits contribute to the decision-making process [80]; the existing research indicates that the public sector often increases the return of investment to enhance the attractiveness of PPP projects for the private sector [81]. Regarding the healthcare domain, most private enterprises participate in healthcare service delivery for profit as emphasised by Mackintosh and his colleagues [82]. The main concern of private equity investors involved in PPP hospitals was reliable revenue [83]. In CHM, nonpublic healthcare services tend to be provided in large cities owing to the considerable consuming market [84]. In addition, Chinese scholars claimed that the overemphasis on public welfare could frustrate the intention of private sector investment in CHM via PPP. Therefore, the following hypothesis is stated.

**Hypothesis 1 (H1).**

AB has significant positive effects on BI.

SN is referred to as the behavioural perception of a decision-maker from important peers or groups [78]. In CHM, the public sector (governments and public hospitals), consumers, and the private sector (private hospital, construction companies pharmaceutical companies, real estate developers, and asset management companies) are three typical stakeholders whose preference is crucial to each participant's behaviour [4]. Ou and Jia stated that the private sector decides whether to participate in Chinese healthcare service delivery via PPP partly in consideration of social image and public reputation [17]. Furthermore, researchers concluded that a positive correlation exists between SN and BI [85]. China PPP Center reported a booming trend in CHM; that is, the number of commercial PPP projects increased from 150 to 171 in two months [86, 87]. Considering that the selection of investment objective from peers could provide an indirect suggestion into the decision-making process of the private sector [65], the imitation effect by former private investors in CHM has influenced others' future behaviour. Thus, the following hypothesis is stated.

**Hypothesis 2 (H2).**

SN has significant positive effects on BI.

PBC is a perception, which reflects the resources and barriers of an expected behaviour [78]. It exhibits the confidence of controlling the resource, which is needed by the private capital. Wang and Zhang indicated that PPP has become a component of China's medical reform; several available approaches have emerged as references for private sector investment in CHM [15]. To improve attractiveness, multiple barriers should be removed to establish an easy access by NHC, whilst private investors participate in elderly care and healthcare service delivery, including free provision for certain procedures [88]. Existing research emphasises that a reduced perceived impediment increases the willingness to generate [74]. Therefore, the following hypothesis is stated.

**Hypothesis 3 (H3).**

PBC has significant positive effects on BI.

FC could be defined as objective factors in an environment to support individual's actual behaviour [89]. Favourable measures in a situational context from organisational support could motivate a positive belief in BI [90]. Certain opportunities with potential benefits provided by public authorities contribute to the participation in social infrastructure delivery of private capital, including extra subsidies, financial support, and land acquiring priority [91]. A large capital and technical rigor exist in the healthcare field; compared with full privatisation with high requirements on financial capacity and operational performance [9], PPP could be regarded as an available method to be involved in Chinese healthcare service delivery. Private capital has been largely encouraged by this favourable circumstance [92]. Thus, the following hypothesis is stated.

**Hypothesis 4 (H4).**

FC has significant positive effects on BI.

#### 3.2.2. Determinants of the Private Sector's Behaviour to Participate in CHM via PPP

General theory believes that BI is determined by AB, SN, and PBC. This study brings FC as a complementary variable in the modified model. The framework based on TPB could be used on investment behaviour to explain investors' decision-making process [72]. Intention is the closest determinant of behaviour [85]. Davies et al. considered that BI maintains a significantly positive influence on usage [93]. Therefore, a strong BI raises the possibility of the actual behaviour to be performed [65]. Thus, the following hypothesis is stated.

**Hypothesis 5 (H5).**

BI has significant positive effects on behaviour.

## 4. Research Method

### 4.1. CB-SEM

CB-SEM is used in this study. This method was first adopted in the social sciences in 1970 and has been extensively applied in marketing, business, management, and behaviour research to identify the connections among certain factors [94, 95]. In addition, several researchers often consider that SEM is as equal as CB-SEM [96]. However, PLS-SEM is another unique approach of SEM. Compared with PLS-SEM, CB-SEM should be generated with a high sample size [97]. Moreover, the objective of CB-SEM is confirmation rather than prediction of PLS-SEM [98]. TPB has been developed for decades with solid basis in theory. CB-SEM could provide more robust estimations compared with PLS-SEM. Therefore, Amos 21.0 is adopted in our research to implement CB-SEM.

### 4.2. Questionnaire

To collect first-hand information, a questionnaire survey was formed containing six latent variables, AB, SN, PBC, FC, BI, and behaviour. The respondents were asked to reply using a five-point Likert scale from 1 (strongly disagree) to 5 (strongly agree). Then, we invited six experts from the field of public health management and four scholars associated with PPP to test the reasonableness and comprehensiveness of the questionnaire. Few items were modified on the basis of the corresponding suggestions from experts and scholars.  Table 2 presents the final questionnaire.

### 4.3. Data Collection

At the project level, the procedure of PPP healthcare project comprises construction (or renewal), operation, and financing. Thus, six groups were selected based on distinct stages: construction companies, real estate developers, pharmaceutical companies, private hospitals, asset management companies, and medical industry property investment companies. Every group has become the stakeholder by getting involved in CHM via different types of PPP (Figure 1).

Considering that the majority of projects in this round of PPP tide were executed contain the construction activity, about a quarter of the respondents were selected from construction companies related to the preoperational stage in this research. Then, pharmaceutical companies, private hospitals, and asset management companies were identified as the participators who hold dominant prevalence of expertise and capacity in operational link. Meanwhile, medical industry property investment companies and real estate developers were considered as the private investors with commercial issues in the financing stage.

The criteria for selecting the sample respondents were as follows: (1) they have expertise in their companies that participate in completed PPP or normal healthcare services; (2) they come from multiple positions and departments; and (3) they are willing to participate in the survey. Therefore, 202 questionnaires were distributed online (WeChat), and 83 were distributed on the spot. All respondents were identified with working experience from the selected occupations. This data collection process lasted for four months from November 2018 to March 2019. Finally, 248 questionnaires were confirmed to be available for this research by removing several questionnaires with low completion or perfunctory effort. Thus, the effective rate is 87%, which could meet the requirement of CB-SEM.  Table 3 lists the demographic data of survey sample.

## 5. Result Analysis

### 5.1. Reliability and Validity

Data reliability and validity should be tested before implementing CB-SEM. SPSS 20.0 software was applied to calculate the Cronbach' *α* value, which reflects the consistency level of the items. Cronbach' *α* value should be more than 0.7 [107]. In this study, the value scale ranges from 0.817 to 0.909; thus, the reliability of the questionnaire could be satisfied. Then, we used Amos 21.0 software to calculate average variance extracted (AVE) value for testing the validity. Fornell and Larcker believed that the AVE value should be more than 0.5 [108]. The six latent variables shown in Table 2 meet the standard, thereby confirming the excellent validity of our research.

### 5.2. Structural Model

After building the structural model, we used Amos 21.0 to test the goodness-of-fit. Multiple indices are connected with this procedure (Table 4). The fit value between our structural model and data meets the acceptable standard.

In addition, composite reliability (CR) was applied to test the internal consistency of the structural model. Hair indicated that the acceptable threshold should be 0.7 [109]; however, Fornell and Larcker believed that 0.6 could also be satisfactory as a standard [108]. In this research, the CR values of all latent variables surpass 0.82 (Table 5).

**Figure 2:**
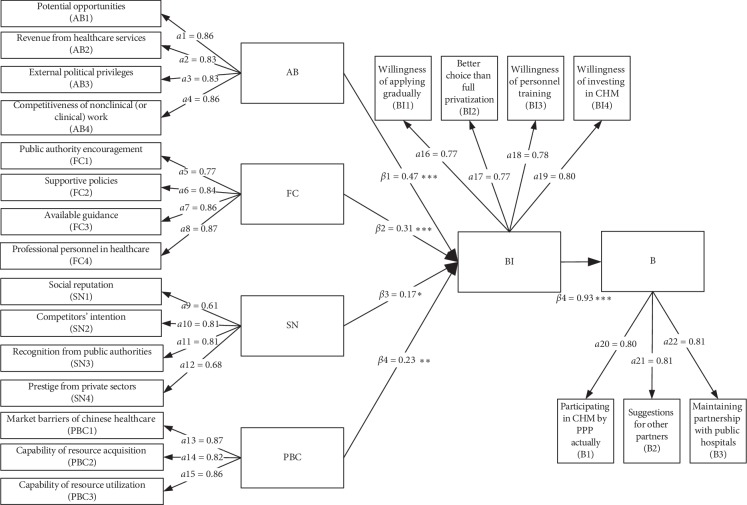
Structural model with path coefficients (*β*) and factor loading (a).

Figure 2 shows the finding of structural model on the private sector's intention and behaviours regarding participation in Chinese healthcare delivery via PPP. Each path among the latent variables corresponds to a certain hypothesis; the six *β* values display the path coefficients (standard regression weights), which are positively correlated with the effect degree. Moreover, we should verify if the hypotheses are supported by calculating the critical ratio (CR) and the *P* value. When the CR is higher than 1.96, the *P* value should be less than 0.05. This finding implies that the path is significant at the 0.05 level, and the hypothesis is supported.  Table 6 presents all the hypotheses' testing results.

As seen from Table 6, the *P* value of all the five paths are less than 0.05, including “AB” to “BI” (*β* = 0.466, *P* < 0.001), “FC” to “BI” (*β* = 0.305, *P* < 0.001), “SN” to “BI” (*β* = 0.167, *P* < 0.05), “PBC” (*β* = 0.231, *P* < 0.01), and “BI” to “B” (*β* = 0.931, *P* < 0.001). These five paths are statistically significant and thus support H1 to H5.

## 6. Discussion

This research established an analytical framework based on modified TPB to determine the influence and relative contribution among certain independent variables for the private sector's intention and behaviour towards participating in CHM via PPP.

Conventional theory suggests that path coefficient was based on standardised regression data [110]; thus, it could be used to compare the relative importance of the independent variables in OLS regression [95]. The degree of four latent variables' contribution to BI can be identified from this perspective.

AB has a significant positive effect on BI with the standard path coefficient of 0.466. This effect could show the closest linkage between attitude and intention in the decision-making process of private capital involvement in CHM via PPP. AB is conducted directly by the feeling on performance of target behaviour [111]. The need for medical resource supply is literally urgent in China given the challenges of ageing, increasing prevalence of chronic diseases, and incompleteness of healthcare delivery [112]. Alternately, Wang and Zhang pointed out that CHM contains several opportunities for the private sector, where most individuals are willing to pay for PPP healthcare services at a reasonable level [15]. Pharmaceutical corporations and medical industry property investment companies are provided huge potential and market volume in China to build large-scale clinical trials of new medicines, devices, and surgical procedures via PPP [113]. In addition, real estate developers attempt to enter into CHM, such as Vanke, China Resources, and Tahoe and Wanda [114]. Several embraced motivation to invest in healthcare service delivery and subsequently obtain an easy access to land acquisition or integrate medical facilities to increase added residential value. Therefore, direct incentives were provided to the private sector, establishing a positive attitude towards participating in CHM via PPP.

FC holds the second strongest significant effect on BI has been verified, with the standard path coefficient of 0.305 in our findings. External facilitators directly contribute to BI, such as technical support and favourable CHM policies [115]. Since Premier Li Keqiang solemnly emphasised the necessity of promoting private capital into CHM [116], numerous supportive policies have been approved, including the expansion of market space, broadening the reimbursement coverage of nonpublic hospitals, and tax relief on primary healthcare facilities by PPP [117]. Furthermore, new technical fields are encouraged to initiate a transformation from outsource contract to PPP, such as telemedicine and E-health system [113]. The internal facilitators are equally critical. Participation in CHM could be highly viable for those private sector entities with experienced personnel, financing channels, and advanced management framework. An increasing number of public hospitals tend to share human resource and medical devices with eligible private sector entities [17]. Evidence from “Beijing Children's Hospital–New Century International Children's Hospital” PPP project confirms this availability.

PBC is positively significant to BI, with a relatively low path coefficient of 0.23. Zheng et al. researched on the willingness of private capital to engage in sustainable performance in PPP infrastructure and confirmed that PBC holds a positive effect on BI [74]. In this study, PBC is also significant to specific intention. The confidence of risk response on specific behaviour directly contributes to BI of participating in CHM. PPP contains more feasibility than traditional public procurement owing to the efficiency-oriented governance structure [118], which could strengthen the confidence and decrease the uncertainty of private capital participation in Chinese healthcare delivery. SN also has a significant effect on BI, with the lowest path coefficient of 0.167, which shows a limited effect. Previous findings suggested that SN holds a strong influence on BI in the decision-making process of the private sector regarding socially responsible investment [72]. The evidence shows a reverse result in our research; the size of standardised direct effect of SN on BI is approximately 1/3 times that of AB. Considering that social reputation is regarded as one of the key factors in hospital selection of patients and most of the nonpublic medical facilities hold unsatisfied prestige in CHM [119], improving the condition in a short time is difficult. Thus, public reputation and social image were deemed as subordinate targets behind business benefit for several private entities in CHM. To a certain extent, these factors cause the lowest effect from SN to BI.

Behaviour is significantly influenced by BI, with the highest path coefficient of 0.931. Numerous studies repeatedly confirmed this close linkage between BI and actual behaviour [120, 121]. Although several findings showed that BI holds a relatively lower effect on behaviour than with our result, it would be totally acceptable considering the difference of context in various studies.

## 7. Conclusions and Implications

Previous studies have been conducted regarding the willingness of the private sector to invest in economic infrastructure via PPP. However, the critical factors related to intention and behaviour of private sector participation in CHM via PPP and the relative importance to which AB, FC, PBC, and SN affect BI were neglected in the relevant literature. This study's innovation is the analysis of the influencing factors of BI and the identification of the level of effect on BI based on modified TPB and CB-SEM. The finding shows that AB, FC, PBC, and SN are positively significant to BI, and that BI has a significant effect on behaviour. Moreover, AB and FC hold a relatively stronger linkage to BI than with others, which provides reference for governments and public authorities to implement appropriate policies for stimulating the private sector's motivation to participate in CHM via PPP and subsequently narrow down the gap of medical resources and ameliorate the quality of Chinese healthcare services.

From this research, the importance of prospective benefits and supportive measures is emphasised. Public authorities should provide official guidelines for private capital involvement in CHM, which could effectively decrease the transaction cost in information searching and contract signing. Thus, additional analysis is made from these two perspectives.

On the one hand, most PPP healthcare projects were newly built, and the integration of stock facilities was less applied. BOT concession could promote the enthusiasm of private capital participation in CHM, considering that several private sector companies in CHM are from the construction industry [122]. However, a long-term institutional inertia of the BT in China causes the construction stage to be a major source of profit and private participants to be scarcely motivated to accept other types of PPP. Therefore, the private entities should be encouraged to establish partnership with public authorities in TOT, ROT, and OM through integration, transformation, or leasing of existing healthcare facilities. The financing risks of these types are distinctly lower than BOT, and private investors might broaden the scope of cooperation and market in a new approach. The Second Social Welfare Center of Beijing has successfully upgraded and integrated medical resources in ROT, which achieved the value between the public and private. In addition, the social sector should be encouraged to participate in CHM in the form of consortium, which could magnify the advantages of construction enterprises in financing, construction, and external relations, as well as the strength of medical industry property investment companies and private hospitals in service performance.

On the other hand, considering the knowledge rigor of the medical field, private sector faces considerable uncertainty in CHM. A standard and feasible exit mechanism should be established. No official guideline exists as a reference for the private sector in exiting PPP. Existing common approaches have obvious limitations in effect, such as expired handover, equity transfer, public listing, bond issuance, and asset-backed securities. We suggest that restrictions on equity transfer of special purpose vehicle could be appropriately liberalised in the Chinese healthcare field. Although governments and public agencies should not be the subject of equity transfer, they may properly allow equity flows among various private capital entities in CHM. To a certain extent, equity flow would help in dispelling the misgivings of the investors and strengthen the intention.

This study contains certain limitations despite its usefulness on the intention and behaviour research of private sector involvement in CHM via PPP. Most of the data from our selected respondents are provided from the questionnaire. Additional studies could be generated via face-to-face interview to acquire highly accurate information and strong explanation.

## Figures and Tables

**Table 1:** Representative types for private sector investment in CHM via PPP.

Types of PPP in the healthcare field	Features	Categories of private sector	Empirical examples
BOT	This type has the widest scope of contract. In practice, the partnership concentrates on the construction of healthcare services without depth in management and operational stages.	(1) Construction companies (2) Real estate developers	(1) Jinan Zhangqiu North Medical Comprehensive Service Center (2) Xingyang People's Hospital (3) Kunming Children's Hospital

TOT	This type is generally applied in existing medical services. Private sector undertakes all business management aspects (drug and medical equipment supply, supermarket, canteen, and property), except core medical care (clinical work).	(1) Pharmaceutical companies (2) Private hospitals (3)Asset management companies	(1) Tongliao Zhongmeng Hospital (2) Nantong Rudong TCM Hospital

ROT	This type is also known as invest-operate-transfer (IOT). Private sector rebuilds and upgrades several equipment based on TOT.	(1) Medical industry property investment companies (2) Construction companies	(1) Shenyang Fifth People's Hospital (2) Hainan Ledong second People's Hospital

O&M	Governments remain the owner of public hospital and outsource nonclinical work (daily operation and management) to the private sector.	(1) Private hospitals (2)Medical industry property investment companies	(1) Beijing Mentougou Hospital (2) Qiannan Huishui Grade-A Tertiary Hospital

**Table 2:** Survey items and certain indices.

Variables and survey items	Cronbach's *α*	AVE	Literature
AB:			
AB1: I could obtain potential opportunities of investment by participating in CHM.	0.909	0.719	[71, 99, 100]
AB2: Participating in CHM could increase operational benefits.			
AB3: Participating in CHM could obtain external political privileges.			
AB4: I can strengthen the competitiveness of nonclinical (or clinical) work by establishing partnership with public authorities.			

SN:			
SN1: Participating in Chinese healthcare service delivery could earn additional social reputation.	0.817	0.537	[74, 101]
SN2: An increasing number of my competitors is involved in CHM.			
SN3: Investing in CHM helps me win recognition from public authorities.			
SN4: Investing in CHM helps me earn prestige from my competitors.			

PBC:			
PBC1: The barriers to enter CHM is acceptable for me.	0.885	0.722	[102, 103]
PBC2: I could gain requisite knowledge and capital to invest in Chinese medical service delivery if I am willing.			
PBC3: Given the necessary resources and knowledge, involvement in CHM would be easy for me.			

FC:			
FC1: Public authorities encourage private investors to participate in CHM.	0.899	0.735	[78, 104, 105]
FC2: Supportive polices with potential benefits have been issued to make an easy approach for private capital investment in CHM.			
FC3: Official guidance is available to me to establish partnership with public authorities and invest in CHM.			
FC4: Employees in my company have received necessary training towards participating in Chinese healthcare service delivery.			

BI:			
BI1: I am willing to try to participate in CHM step by step.	0.865	0.607	[71, 106]
BI2: Investing in CHM by PPP rather than other fields or full privatisation is a good idea.			
BI3: Personnel in my company would be glad to accept related training.			
BI4: CHM would be one of my favourite fields to invest by PPP.			

B:			
B1: I have engaged in Chinese healthcare resources supply via PPP.	0.847	0.649	[78]
B2: I suggested other partners to also participate in CHM via PPP.			
B3: I will keep partnering with public authorities and investing in CHM.			

**Table 3:** Sample demographics.

	Type	Frequency	Percentage (%)
Gender	Male	166	66.9
	Female	82	33.1

Working experience	3–5 years	53	21.4
	6–10 years	122	49.2
	11–15 years	31	12.5
	16–20 years	29	11.7
	More than 20 years	13	5.2

Occupation	Construction companies	64	25.8
	Real estate developers	53	21.4
	Pharmaceutical companies	27	10.9
	Private hospitals	39	15.7
	Asset management companies	41	16.5
	Medical industry property investment companies	24	9.7

Position	Administrative staff	173	69.8
	Basic staff	75	30.2

**Table 4:** Goodness-of-fit test measure.

Goodness-of-fit measure	Acceptable threshold	Value of model	Evaluation
CMIN/DF	<3.00	2.005	√
GFI	>0.90	0.906	√
AGFI	>0.90	0.886	√
RFI	>0.90	0.911	√
NFI	>0.90	0.936	√
CFI	>0.90	0.950	√
RMSEA	<0.08	0.064	√
PNFI	>0.50	0.777	√
PCFI	>0.50	0.815	√
PGFI	>0.50	0.686	√

**Table 5:** CR value of latent variables.

Latent variables	Composite reliability value
AB	0.911
FC	0.917
SN	0.820
PBC	0.886
BI	0.861
B	0.847

**Table 6:** Hypothesis assessments.

Hypothesis	Path	*β*	CR (*t*)	*P*	Result
H1	BI ⟵ AB	0.466	8.712	^*∗∗∗*^	Supported
H2	BI ⟵ FC	0.305	5.999	^*∗∗∗*^	Supported
H3	BI ⟵ SN	0.167	2.466	^*∗*^	Supported
H4	BI ⟵ PBC	0.231	3.136	^*∗∗*^	Supported
H5	B ⟵ BI	0.931	12.588	^*∗∗∗*^	Supported

^*∗∗∗*^ *P* < 0.001; ^*∗∗*^*P* < 0.01; ^*∗*^*P* < 0.05.

## Data Availability

The data used to support the findings of this study are available from the corresponding author upon reasonable request.
